# Mechanisms and environmental factors shaping the ecosystem of brain macrophages

**DOI:** 10.3389/fimmu.2025.1539988

**Published:** 2025-01-24

**Authors:** Silvia Penati, Simone Brioschi, Zhangying Cai, Claudia Z. Han, Marco Colonna

**Affiliations:** ^1^ Department of Pathology and Immunology, Washington University School of Medicine in Saint Louis, Saint Louis, MO, United States; ^2^ Brain Immunology and Glia (BIG) Center, Washington University School of Medicine in Saint Louis, Saint Louis, MO, United States

**Keywords:** brain macrophages, microglia, border-associated-macrophages, ontogeny, yolk sac, brain development

## Abstract

Brain macrophages encompass two major populations: microglia in the parenchyma and border-associated macrophages (BAMs) in the extra-parenchymal compartments. These cells play crucial roles in maintaining brain homeostasis and immune surveillance. Microglia and BAMs are phenotypically and epigenetically distinct and exhibit highly specialized functions tailored to their environmental niches. Intriguingly, recent studies have shown that both microglia and BAMs originate from the same myeloid progenitor during yolk sac hematopoiesis, but their developmental fates diverge within the brain. Several works have partially unveiled the mechanisms orchestrating the development of microglia and BAMs in both mice and humans; however, many questions remain unanswered. Defining the molecular underpinnings controlling the transcriptional and epigenetic programs of microglia and BAMs is one of the upcoming challenges for the field. In this review, we outline current knowledge on ontogeny, phenotypic diversity, and the factors shaping the ecosystem of brain macrophages. We discuss insights garnered from human studies, highlighting similarities and differences compared to mice. Lastly, we address current research gaps and potential future directions in the field. Understanding how brain macrophages communicate with their local environment and how the tissue instructs their developmental trajectories and functional features is essential to fully comprehend brain physiology in homeostasis and disease.

## 1 Introduction

Macrophages are innate immune cells serving as professional phagocytes within body tissues (1, 2). Phagocytes are evolutionarily ancient and have been described in multiple animal phyla, including arthropods, echinoderms, mollusks, and worms (3). Thus, macrophages (and their cellular ancestors) may represent one of the first cell-mediated defenses during the evolution of the immune system. Phagocytic cells were first discovered by the Russian zoologist Élie Metchnikoff between 1882 and 1884 during his studies on the immune responses in frogs and starfish. Macrophages were described amoeboid cells capable of migrating to sites of injury and engulfing pathogens, dead cells, or foreign material (4, 5). Today, over nearly half a century after their discovery, macrophages remain a topic of extensive immunological and biomedical research. We now know that these cells not only coordinate immune responses during injury or infection, but they are also essential for organ development, homeostasis, and tissue repair.

Like any other organ, the central nervous system (CNS) harbors its own population of resident macrophages. Recent studies have demonstrated that CNS macrophages encompass highly heterogeneous populations, with distinct spatial distributions and immunological features. Microglia are the resident macrophages of the CNS parenchyma, while border-associated macrophages (BAMs) are located at the CNS interfaces, such as meninges, ventricles and perivascular compartments. Both microglia and BAMs originate from primitive myeloid progenitors during embryonic hematopoiesis, but they undergo divergent fates within the CNS environment. Microglia and BAMs specification trajectories differentially shape their phenotypes and functions throughout development, thus generating distinct macrophage populations that are highly specialized for their CNS microenvironments. The scope of this review is to provide a comprehensive yet succinct overview of the developmental mechanisms of CNS macrophages to non-specialists. Here, we discuss the current understanding of these cells, emphasizing the latest findings on their phenotypic diversity, transcriptional and epigenetic programs, and communications pathways within their tissue niches. We also discuss and contextualize emerging findings from human studies. Lastly, we address current knowledge gaps and outstanding questions that warrant further investigation in the years to come.

## 2 History of microglia research

The pioneering application of microscopy techniques to study biological systems is historically accredited to the Dutch microbiologist Antonie van Leeuwenhoek, who in 1676 observed motile microorganisms using a homemade optical microscope of his invention (6). Further optimization of lens microscopes and tissue staining methods, especially silver-nitrate staining, allowed Nobel laureates Camillo Golgi and Santiago Ramón y Cajal to describe the arborized morphology of brain cells in the late 19th century. These cells were later named “neurons” and recognized as the functional unit of the nervous system. Because neurons were the first brain cells to be discovered, neuropathologists referred to them as the brain’s “first element”. The term “glia” or “neuroglia” (from the Greek “glue”) was introduced by Rudolf Virchow in 1856, indicating an amorphous matter filling the gaps between brain cells and providing structural support to the nerve tissue (7). Through his studies, Ramón Cajal realized that the glia described by Virchow were indeed a distinct population of brain cells, separate from neurons, and they were then termed “glial cells” or the “second element” (y 8). The word “astrocyte” (from the Greek “star cell”) was introduced in 1895 by the Hungarian neuroanatomist Michael von Lenhossék, inspired by the stellate morphology of these cells (9, 10).

Between the 19th and 20th centuries, neuropathologists Franz Nissl and Alois Alzheimer applied optimized staining techniques in their histological analyses of the human brain and noted the presence of an unknown cell type, distinct from neurons and astrocytes (11). These cells were characterized by an amoeboid cell body and fine cellular processes and were described as brain phagocytes that accumulated in diseased brains, particularly near lesion sites such as amyloid plaques. Cajal designated these cells as the brain’s “third element,” while William Ford Robertson introduced the term “mesoglia” to emphasize their distinct nature compared to neuroglia (12).

The seminal characterization of microglia was performed by Cajal’s alumnus, Pio del Rio-Hortega, between 1919 and 1928 (13). The Spanish neuroscientist utilized a modified version of the silver carbonate staining method, which led to the classification of two new brain cell types: microglia and oligodendrocytes. Del Rio-Hortega himself introduced the term “microgliocytes” or “microglia” to distinguish them from larger glial cells such as astrocytes and oligodendrocytes (collectively known as “macroglia”). According to del Rio-Hortega’s observations, microglia are glial cells of mesodermal origin that presumably infiltrate the brain through the pia mater during early development. He described adult microglia as having a ramified morphology when in a resting state, with processes extending from the cell body. Notably, del Río-Hortega also recognized that, in response to disease or injury, microglia become amoeboid, migrate toward the site of damage, proliferate, and engage in phagocytosis to clear cellular debris; a description that remains accurate today (14–16).

Microglia research remained relatively quiescent until the late 1960s, when the German neuropathologist Georg W. Kreutzberg inspected brain stem microglia using electron microscopy in a rodent model of facial nerve axotomy (17). The ultrastructural resolution allowed Kreutzberg to highlight the physical interaction between microglia and neuronal synapses, showing that microglia could engulf and remove synapses from damaged neurons (17). This finding revealed, for the first time, mechanisms of microglia-neurons communication. However, it was in the 1990s that microglia research led to fundamental discoveries in the field, such as the introduction of microglia cell culture techniques (18), electrophysiological recording of ion currents in microglia (19–21), and the optimization of staining strategies to study microglia in flow cytometry (22) and histology (23, 24).

Finally, the generation of the Cx3cr1-GFP line (25), which induces GFP expression in CX3CR1+ cells, including microglia, monocytes, and BAMs, revolutionized the ability to study microglia dynamics in vivo. For instance, this mouse strain enabled the visualization of yolk-sac derived macrophages accumulating in the cephalic mesenchyme and penetrating the neuroepithelium at embryonic days E10.5 (26), providing direct evidence of the embryonic origin of microglia. Using in vivo two-photon microscopy, researchers observed microglial processes motility under homeostatic conditions and after brain injury (27, 28). These seminal studies revealed that microglia continuously survey their microenvironment by extending and retracting their processes, establishing brief contacts with neighboring synapses. Furthermore, microglia motility was shown to be driven by neuronal activity and ATP signaling through the microglial P2Y receptors (27–29). More recently, the generation of the constitutive Cx3cr1-Cre and the tamoxifen-inducible Cx3cr1-CreErt2 mouse lines has allowed genetic manipulation of microglia in vivo (30, 31) broadening opportunities for functional studies.

## 3 Microglia originate from yolk sac myeloid progenitors

### 3.1 Early studies

Since the discovery of microglia by del Rio-Hortega and extending into the first half of the 20th century, the origin of tissue phagocytes, including microglia, have long been debated. In 1968, van Furth and Cohn proposed a model known as “the mononuclear phagocyte system” (MPS) asserting that bone marrow (BM) progenitors give rise to circulating monocytes, which then differentiate into macrophages upon extravasation into the body tissues (32–34). Although this theory became dogmatic in immunology textbooks, some experimental evidence challenged its universal validity (35). For example, hematopoietic progenitors generating macrophages were found in the yolk sac before the onset of definitive hematopoiesis in the embryo proper (36–38). Furthermore, yolk sac progenitors exhibited mostly myeloid/erythroid potential compared to the definitive hematopoietic stem cells (HSCs) that emerged in the paraaortic splanchnopleura of the dorsal aorta before migrating into the fetal liver (39–44), suggesting that the two hematopoietic waves are temporally, spatially and ontogenically unrelated (45–47). Nevertheless, BM transplantation studies using irradiation or chemotherapeutic regimen observed infiltration of monocyte-derived macrophages in the host brain parenchyma, generating a long-lasting controversy on the possible BM origin of microglia (48, 49). However, later studies unambiguously demonstrated that BM transplantation strategies induce blood-brain-barrier damage thus eliciting artificial monocytes recruitment (50).

### 3.2 The revolution of fate-mapping strategies

**Figure 1:**
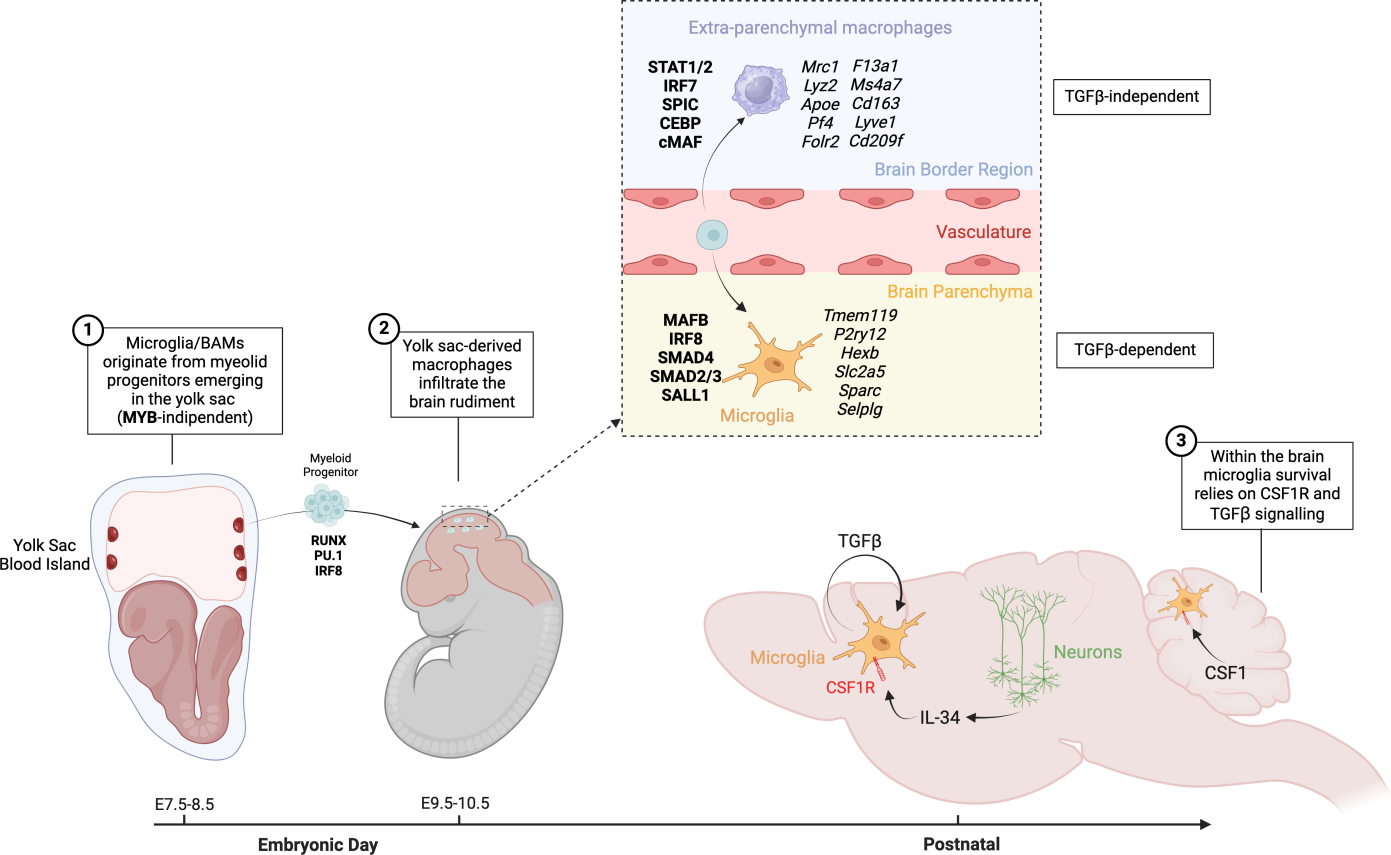
Origin and development of microglia and BAMs. Microglia and BAMs originate from myeloid progenitors emerging in the yolk sac between embryonic days E7.5 and E8.5, in a process that is MYB-independent. Around E9.5 to E10.5, yolk sac-derived macrophages infiltrate the brain rudiment. Depending on the local niche, these infiltrating cells differentiate into either BAMs or microglia. BAMs develop in a TGFβ-independent manner, whereas the differentiation of microglia is dependent on TGFβ signaling. In the brain, microglia rely on CSF1 signaling for their maintenance and function.

The advent of genetic fate-mapping tools provided definitive proof that microglia originate from erythro-myeloid progenitors (EMPs) emerging in the yolk sac between E7.5-E8.5 (**Figure 1**), with no significant contribution from fetal liver or BM hematopoiesis (26, 51–54). Unlike HSCs, yolk sac EMPs, a di-potent progenitor also capable of generating primitive erythrocytes, produce macrophages in a MYB-independent manner and without a monocytic intermediate stage (52, 55, 56). Instead, EMPs differentiate into committed myeloid progenitors, macrophage precursors and eventually macrophages upon activation of a myeloid transcriptional program that relies on the transcription factors RUNX1, PU.1 and IRF8 (57, 58). These yolk sac-derived macrophages infiltrate the brain rudiment around E9.5-10.5, after embryonic circulation is established, and serve as the primary source of microglia (26, 59) (**Figure 1**). Indeed, Ncx1-deficient embryos, which have impaired circulation, lacked brain microglia while preserving yolk sac myelopoiesis (26). Lineage tracing approaches also confirmed that under homeostatic conditions microglia are maintained by slow self-renewal throughout the mouse lifespan with negligible input from BM hematopoiesis; a property shared with other tissue-resident macrophages of embryonic origin, such as Kupffer cells of the liver, and Langerhans cells in the epidermis (30, 50, 51, 60–67).

## 4 Trophic factors for microglia

### 4.1 CSF1R signaling is required for microglia survival

Microglia survival critically depends on Colony Stimulating Factor 1 Receptor (CSF1R) signaling. Genetic deletion of Csf1r (26, 68, 69) or CSF1R blockade using antagonist antibodies or pharmacological inhibitors (70, 71) leads to complete microglia depletion. Similarly, knock-out mice for the fms-intronic regulatory element (FIRE), a super-enhancer in the second intron of the Csf1r locus, are completely devoid of microglia (72), while preserving most of the peripheral macrophage populations. The importance of CSF1R signaling is further underscored by its role in human neurological diseases. Individuals carrying CSF1R loss-of-function mutations develop a series of neurological conditions such as adult-onset leukoencephalopathy with axonal spheroids and pigmented glia (ALSP), or hereditary diffuse leukoencephalopathy with spheroids (HDLS), brain abnormalities, neurodegeneration, and dysosteosclerosis (BANDDOS) (73–77). Aged FIRE mice also developed ALSP-related features, such as myelin degeneration and disruption of cholesterol metabolism (78). These findings highlight the importance of CSF1R signaling in microglia to maintain CNS homeostasis.

Interestingly, CSF1-deficient (Csf1op/op) mice only exhibit about a 30% reduction in microglia (26), suggesting that other CSF1R ligands may play compensatory roles for microglia survival. Indeed, the identification of the cytokine IL34, the second CSF1R ligand, was a fundamental discovery for the microglia field (79). IL34 and CSF1 are analogous cytokines that share a similar 3D structure despite having little sequence homology, suggesting evolutionary convergence (80, 81). IL34 is highly expressed in neurons, especially in gray matter where CSF1 production is scarce. Indeed, Il34 knock-out mice display a significant microglia deficiency in forebrain regions such as the cortex, hippocampus, and striatum (69, 82–84). Conversely, IL34 is not expressed in the cerebellum, where CSF1 is abundant. Thus, Csf1 deletion in neuroectodermal cells results in microglia depletion from cerebellar gray matter, while forebrain microglia remain unaffected (84–86). Consistently, the inhibition of either CSF1 or IL34 with blocking antibodies depleted microglia in distinct brain regions. Specifically, anti-CSF1 selectively depleted microglia in white matter regions, such as the corpus callosum, hippocampal fimbria, and striatal white matter, whereas anti-IL34 primarily affected gray matter microglia in the cortex and striatum (87). Overall, CSF1 appears critical for microglia survival in white matter, while IL34 mostly maintains microglia in gray matter.

In addition to their spatial specificity, IL34 and CSF1 also differ in their temporal expression patterns. IL34 levels in the brain peak during the perinatal period and decline during adulthood (82). Consistently, the brains of IL34-deficient mice appear normal in E10.5–18.5 embryos (82), while microglia loss starts being observed from postnatal day 2 (83), suggesting that microglia become IL34-dependent after birth only (87). In summary, the regional and temporal specificity of IL34 and CSF1 underscores the non-redundant roles of these cytokines in supporting distinct subpopulations of microglia throughout the brain (69). These findings illustrate how different CNS microenvironments orchestrate heterogeneous communication pathways with microglia.

### 4.2 TGFβ signaling drives microglia development

After reaching the boundaries of the CNS rudiment around E10.5, yolk sac-derived macrophages express high levels of CD206 (Mrc1), while microglia genes are not yet detectable. Differentiation into microglia commences immediately upon their infiltration into the developing brain (88, 89). Once in the parenchyma, embryonic microglia undergo a specification program sculpting their homeostatic phenotype throughout the embryonic and early post-natal period. Along this process, microglia downregulate genes like Mrc1, Lyz2 and Apoe that are highly expressed in extra-parenchymal macrophages and gradually upregulate microglia signature genes like Tmem119, P2ry12, Hexb, Slc2a5, Sparc and Selplg, reaching complete maturity around the third post-natal week (90–94).

Microglia specification is orchestrated by a repertoire of transcription factors like Mafb, Irf8, Smad4 and Sall1 (91, 95–100), as well as by stimuli from the brain parenchymal environment, such as the transforming growth factor beta (TGFβ) signaling (101). Multiple studies have demonstrated that TGFβ crucially instructs the microglia developmental program in macrophages infiltrating the brain parenchyma. Deletion of critical components of the TGFβ signaling cascade, such as the cytokine Tgfb1 (101), the latent-TGFβ binding-proteins Lrrc33/Nrros (102, 103), the integrin activating latent-TGFβ (104), the TGFβ receptors (54, 95, 105, 106), or the downstream transcription factor Smad4 (98, 99) consistently impaired microglia development, holding microglia in an undifferentiated state. More specifically, it has been shown that TGFBR2- or SMAD4-deficient microglia failed to upregulate their homeostatic signature genes, while maintaining expression of genes enriched in CD206+ microglia precursors (Mrc1, Lyz2, Apoe, Pf4, Ms4a7) that accumulate at the borders of the embryonic brain (54, 98). Such developmental failure is phenocopied by the deletion of the microglia-specific Sall1 super-enhancer activated by SMAD4 (99). Transcriptomic and epigenetic analyses demonstrated that SMAD4 and SALL1 cooperate to activate the expression of microglia genes, while repressing genes of extra-parenchymal macrophages (99). Interestingly, microglia are the main source of TGFβ1 in the brain (78), suggesting that their maturation is sustained through an autocrine feedforward loop. Indeed, Tgfβ1 deletion in microglia recapitulated the developmental failure observed after conditional deletion of Tgfbr2 or Smad4 (107, 108). Importantly, postnatal deletion of TGFβ1, TGFβR2 or SMAD4 caused microglia dedifferentiation and reestablished their immature phenotype (99, 107, 108). This data suggests that TGFβ signaling in microglia is not only required to activate the microglia specification program in the embryonic brain, but also to maintain their mature phenotype after birth.

Intriguingly, a similar microglia phenotype has been described in mice with Integrin-β8 (Itgβ8) deletion in neuroectodermal brain cells using Nestin-Cre or Emx1-Cre (104, 107), targeting neuronal stem cells (or radial glia) during brain development. ITGβ8 forms a heterodimer with Integrin-αV (commonly αVβ8) binding the latent-TGFβ complex produced by microglia themselves. This interaction unleashes active TGFβ which engages TGFβ-receptor on microglia in an autocrine manner (109). Thus, radial glia lining the ventricle walls in the embryonic CNS activate the TGFβ signaling in microglia precursors while infiltrating the brain parenchyma during development (107).

Despite being largely consistent, these studies occasionally reported distinct behavioral phenotypes in mice with abrogation of TGFβ signaling in microglia, ranging from neurodevelopmental alterations and neuromotor dysfunctions (104, 107), white matter disease (78, 104) or learning and memory deficits (98, 108). Different behavioral phenotypes may stem from the target gene being deleted. TGFβ or TGFβ-receptors deletion simultaneously abrogates canonical (SMAD4-dependent) and non-canonical (SMAD4-independent) TGFβ pathways. Conversely, deletion of SMAD4 impairs the canonical pathway only, while preserving non-canonical pathways (110). Further studies are required to better understand the exact implications of the different TGFβ pathways in microglia.

In summary, microglia precursors produce latent-TGFβ1, which is activated by radial glia during infiltration into the brain parenchyma. Active TGFβ1 engages TGFβ-receptors on microglia precursors thus promoting a self-sustained SMAD4/SALL1 transcriptional program leading their stepwise maturation throughout the embryonic and early post-natal period.

## 5 Brain extra-parenchymal macrophages

**Figure 2:**
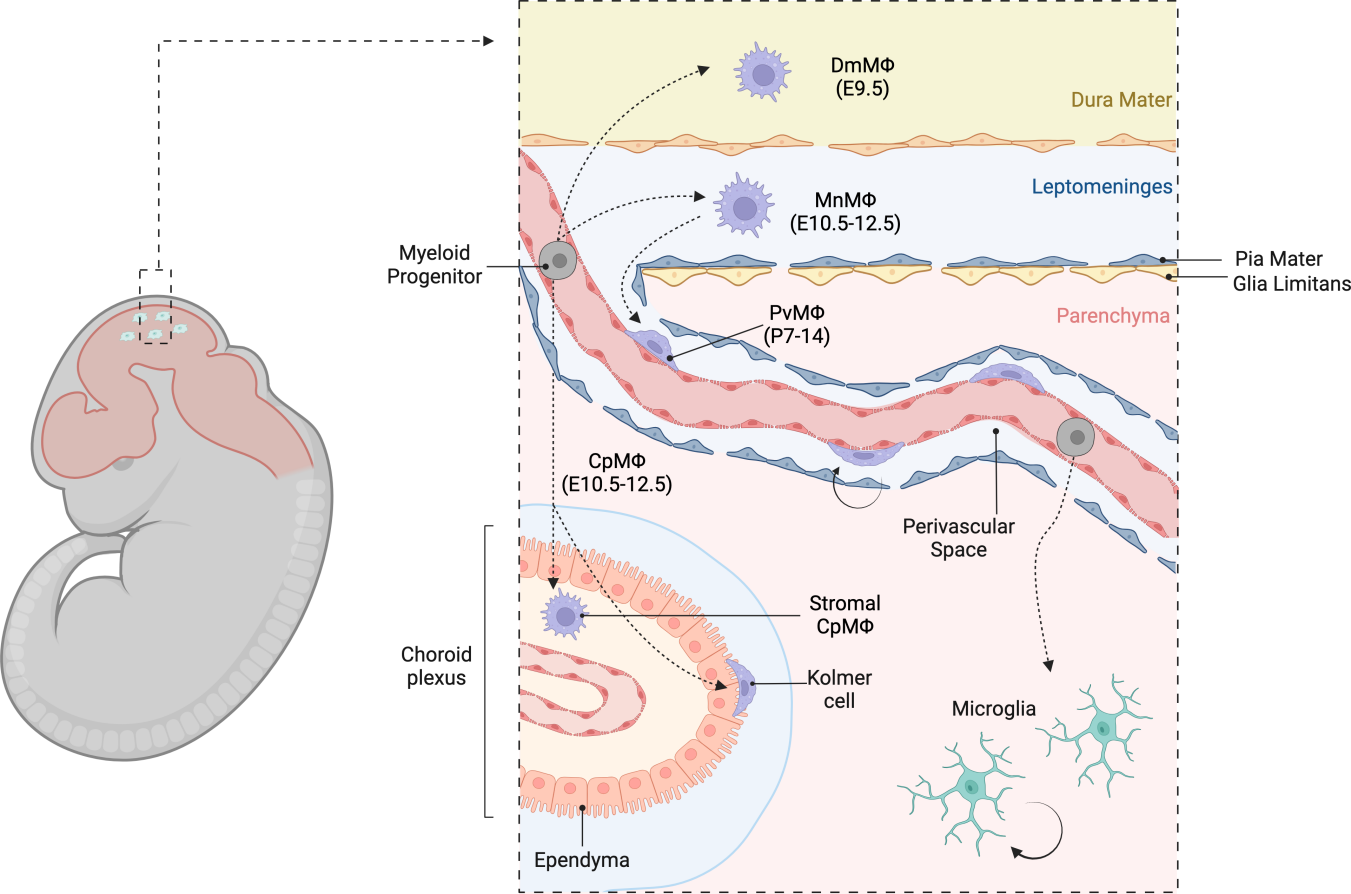
Heterogeneous populations of CNS border macrophages. Diverse populations of extra-parenchymal macrophages in the CNS border regions, emphasizing their spatial and phenotypic heterogeneity. These macrophages can be classified based on their topological distribution into perivascular macrophages (PvMΦ) located in the Virchow- Robin perivascular spaces, meningeal macrophages (MnMΦ) in the leptomeninges, dural macrophages (DmMΦ) in the dura mater, and choroid plexus macrophages (CpMΦ) within the choroid plexus. Collectively, these macrophages are referred to as CNS-associated macrophages (CAMs) or border-associated macrophages (BAMs).

The CNS border regions harbor heterogeneous populations of extra-parenchymal macrophages that are spatially and phenotypically diverse (98, 111, 112). Based on their topological distribution, these cells can be classified into perivascular macrophages (PvMΦ) in the Virchow-Robin perivascular spaces, meningeal macrophages (MnMΦ) in the leptomeninges, dural macrophages (DmMΦ) in the dura mater, and choroid plexus macrophages (CpMΦ) within the choroid plexus (113–115) (**Table 1**). These border-zone macrophages are collectively referred to as CNS-associated macrophages (CAMs) (117) or border-associated macrophages (BAMs) (118). PvMΦ and MnMΦ are the only macrophage subsets that are in direct contact with the brain proper and have been also referred to as subdural macrophages (SDMs) (112) or parenchymal border macrophages (PBMs) (119) (**Figure 2**).

**Table 1:** Location, origin and marker genes of different BAM subsets.

Population	Location	Origin	Markers	References
Meningeal Macrophages (MnMΦ)	Leptomeninges	Yolk sac progenitors and some contribution of BM-derived macrophages after birth	YS origin *Mrc1, Pf4, F13a1, Cd163, Ms4a7, Lyve1, Folr2, Cd209f* HSCs origin *Lyz2, H2-Aa, H2-Ab1, Cd74, Clec12a, Axl, Cd52, Ccr2*	88, 98, 111, 112
Perivascular Macrophages (PvMΦ)	Virchow-Robin perivascular space	Mostly yolk sac progenitors	*Mrc1, Pf4, F13a1, Cd163, Ms4a7, Lyve1, Folr2, Cd209f*	88, 98, 111, 112
Dural Macrophages (DmMΦ)	Dura mater	Yolk sac progenitors. Contribution of BM-derived macrophages increases after birth and during aging.	Two main populations: MHCII^hi^ and MHCII^low^, Incomplete understanding of their phenotypes	112
Stromal Choroid Plexus Macrophages (CpMΦ)	Choroid plexus stroma	Yolk sac progenitors. Contribution of BM-derived macrophages increases after birth and during aging.	Two main populations: MHCII^hi^ and MHCII^low^, *Lilra5*	112–116
Epiplexus Kolmer Cells	Choroid plexus epithelial cell layer facing the ventricle’s lumen	Mostly yolk sac progenitors	Several microglia signature genes: *Trem2, Hexb, Tmem119, P2ry12, Siglech, Slc2a5, Sparc, Sall1*	112

Table summarizing the known characteristics of various BAM populations in the mouse brain.

The underline is used to distinguish markers for meningeal macrophages of diﬀerent origins. Specifically, Mrc1, Pf4, F13a1, Cd163, Ms4a7, Lyve1, Folr2, and Cd209f are markers for macrophages derived from the yolk sac, whereas Lyz2, H2-Aa, H2-Ab1, Cd74, Clec12a, Axl, Cd52, and Ccr2 are markers for macrophages originating from hematopoietic stem cells (HSCs).

### 5.1 Meningeal and perivascular macrophages

Like microglia, virtually all BAM subsets originate from yolk-sac hematopoiesis, with minor contributions from fetal liver or BM monocytes (54, 120). Although microglia and BAMs disseminate the developing CNS at the same time, they undertake completely distinct developmental paths (54, 88). While TGFβ signaling shapes the microglia homeostatic signature throughout the embryonic and early postnatal period, BAMs develop independently from TGFβ (54, 98) and acquire a different repertoire of marker genes (Mrc1, Lyz2, Apoe, Pf4, F13a1, Ms4a7, Cd163, Lyve1, Folr2, Cd209f) (98, 112). Divergent specifications programs in microglia and BAMs are likely controlled by different transcription factors. For example, microglia show stronger transcriptional signatures associated with of IRF8, MAFB, SMAD2/3 and SALL1 transcriptional networks, while BAMs are more enriched for STAT1/2, IRF7, SPIC, CEBP and cMAF (98, 100, 112, 121, 122). Interestingly, both SMAD4 and MAFB are not required for the development of BAMs (98, 112, 121, 122), suggesting that these transcription factors are specifically induced by the parenchymal compartment. The mechanism through which the extra-parenchymal CNS niches instruct the transcriptional trajectory in BAM is still unclear.

The earliest populations colonizing the CNS border regions are MnMΦ and CpMΦ between E10.5-12.5 (88, 116). The perivascular spaces begin to open alongside the large-medium caliber blood vessels of the cerebral cortex early postnatally. Between the first and the second postnatal weeks, MnMΦ migrate along the pia mater penetrating the upper cortical layers, thus generating PvMΦ, which locally expand by cell division (88). Migration of MnMΦ in the perivascular compartment is controlled by integrins and is facilitated by the presence of vascular smooth muscle cells (88); however, the exact mechanism remains to be fully elucidated. In the postnatal brain, PvMΦ are largely MHCII low/negative, expresses high levels of CD206, FOLR2, LYVE1 and CD38, and retain embryonic origin and self-renewal capacity (98, 118, 123). In the leptomeninges, however, at least two distinct populations of MnMΦ can be observed (98, 118, 123). One subset expressing CD206, FOLR2, LYVE1 and CD38 is phenotypically and ontogenically equivalent to PvMΦ. Conversely, a second subset dim or negative for these markers but positive for MHCII is mostly HSCs-derived and is established postnatally (98, 112, 118). A similar phenotypic heterogeneity has been observed in DmMΦ (112, 124). Like other BAM populations, DmMΦ initially originate from yolk sac progenitors, however, they are partially replaced by monocyte-derived macrophages afterbirth. Nevertheless, some DmMΦ of embryonic origin persist throughout adulthood (124). Similar to other CNS border regions, DmMΦ comprise two main populations: one subset expressing high levels of CD206, FOLR and CD301a, and another subset expressing MHCII and low levels of CD206. While CD206hi DmMΦ are initially yolk sac-derived, MHCII+ DmMΦ expand significantly during the first postnatal weeks. Monocyte-derived macrophages contribute to both subsets during development and adulthood, with a stronger input to the MHCII+ population. Indeed, the MHCII+ DmMΦ appear reduced in Ccr2-/- mice, where monocytes are retained in the BM (112, 124).

### 5.2 Choroid plexus macrophages

CpMΦ encompass highly heterogeneous macrophage populations with different phenotypes, origin and spatial distribution (112). The choroid plexus is a ventricular organ that produces cerebrospinal fluid (CSF). It consists of a monolayer of ciliated epithelial cells contiguous with the ventricular ependyma, supported by a stroma containing mesenchymal cells, connective tissue and fenestrated blood vessels. The choroid plexus begins to form around E10.5, to become fully developed at birth (115, 125). Macrophages are highly abundant in this region and generally fall into two main categories: stromal CpMΦ and epiplexus Kolmer cells located on the epithelial layer facing the ventricle’s lumen. Both populations of CpMΦ are established early during embryonic development; however, stromal CpMΦ are gradually replaced by monocyte-derived macrophages during aging (112, 116). Like MnMΦ, stromal CpMΦ can be MHCII+ or MHCII−, though it remains to be determined whether these two subsets represent developmentally and biologically distinct populations rather than transitory activations states (112). Stromal CpMΦ share several marker genes with PvMΦ/MnMΦ (SDMs or PBMs), however there are notable differences (112). PvMΦ/MnMΦ express high levels of Lyve1 and Cd209f, which are not detectable in CpMΦ. Conversely, CpMΦ express the inhibitory receptor Lilra5. These differences may reflect highly specialized functions tailored to the local environment. For example, PvMΦ/MnMΦ might be more prone to interact with vascular components such as hyaluronan (LYVE1 ligand), while CpMΦ are more frequently exposed to immunological challenges and may play a role in the production of CSF molecules.

Epiplexus Kolmer cells are an elusive subset of CpMΦ due to the lack of effective staining techniques for histology or flow cytometry. Consequentially, this CpMΦ population remains highly understudied. Nevertheless, one study reported that Kolmer cells co-express BAM genes (such as Lyz2, Apoe, but low levels of Mrc1), along with several microglia signature genes (Trem2, Hexb, Tmem119, P2ry12, Siglech, Slc2a5, Sparc and Sall1). Like microglia, Kolmer cells appeared long-lived and did not receive significant input from blood monocytes (112). The optimization of staining, sorting and genetic targeting strategies for Kolmer cells is warranted to study this population in greater detail.

### 5.3 Microglia and BAMs have specialized functions in CNS homeostasis and immunity

Microglia are known to play several essential functions to maintain CNS homeostasis. For example, microglia regulate the brain’s physiology by shaping neuronal connectivity (71, 84, 126, 127), remove cellular debris, apoptotic corpses and toxic protein aggregates during brain development, aging and disease (128–131), promote homeostatic myelination and myelin repair after injury (78, 132, 133), and activate healing processes after tissue damage (134–136).

In comparison to microglia, our understanding of the BAMs’ immunobiology remains limited. However, recent seminal studies provided some insight into the functional roles of BAMs under steady state or disease. For example, PvMΦ have been shown to regulate CSF flow through the meninges by modulating extracellular matrix deposition along the CNS endothelial cells (119). Additionally, PvMΦ have been identified as key contributors to neurovascular dysfunction and impaired cerebral blood flow in mice expressing human APOE4 (137). These findings suggest that PvMΦ play key roles in regulating the physiology of brain blood vessels.

During immune responses, BAMs represent the first line of defense against meningeal infections caused by viruses (138–140), bacteria (141, 142), and parasites (143). These studies have demonstrated that BAMs support the recruitment of monocytes and effector T cells, facilitating pathogen clearance. Due to their strategic location at the brain borders, BAMs are able to present antigens to cognate CD4 T cells during CNS autoimmunity or neurodegeneration (144–147). Nevertheless, several studies showed that infiltration and activation of meningeal T cells occurs independently of MHCII expression in BAMs or microglia (111, 124, 139, 144). Instead, BAMs appear to play a major role in cytokines and chemokines secretion through type-I interferon signaling (111, 139). Additionally, DmMΦ have been shown to perform efferocytosis of apoptotic neutrophils in the dura mater during neuroinflammation (124). These findings suggest that BAMs may play a role both in the onset and resolution of inflammatory dynamics within the CNS. Consistently, a study on bacterial meningitis showed that CpMΦ contribute to epithelial barrier repair following neuroinflammation, raising the hypothesis that other BAM subsets, such as MnMΦ and PvMΦ, might also possess similar tissue-healing properties (142).

Taken together, BAMs are emerging as key regulators of vascular dynamics, immune surveillance, and tissue repair within the CNS. Nevertheless, our understanding of these mechanisms remains limited. Further investigations are warranted to explore BAMs as potential therapeutic targets for vascular and neuroinflammatory conditions.

## 6 The CNS environment sculpts the phenotype of microglia and BAMs

### 6.1 The brain parenchyma instructs the microglial phenotype

Several studies demonstrated that the CNS environment crucially instructs the microglial phenotype by shaping the repertoire of tissue-specific enhancers and transcription factors (148–150). To study these mechanisms in vivo, strategies of microglia depletion/repopulation have been employed. Head irradiation induces blood-brain-barrier damage and, to some extent, monocyte infiltration into the brain (50). However, a persistent microglia ablation elicits massive monocyte infiltration into the CNS, even in absence of blood-brain-barrier preconditioning. Multiple groups have utilized genetic models of microglia depletion to promote monocyte infiltration into the brain, thus replacing resident microglia with monocyte-derived macrophages (151–156). Consistently across these studies, monocyte-derived macrophages acquire a phenotype and epigenetic profile largely overlapping with that of endogenous microglia, suggesting that local tissue factors within the brain parenchyma induce the differentiation of monocytes into microglia-like cells. Nonetheless, monocyte-derived microglia-like cells still retain transcriptional and epigenetic traits reminiscent of their BM origin. For example, genes like Apoe, Lyz2, Clec12a or the surface marker F4/80 were consistently upregulated in microglia-like cells compared to endogenous microglia, while Sall1 expression was barely detectable (152, 154, 155). Furthermore, monocyte-derived microglia-like cells mounted more pronounced inflammatory responses during immunological challenges than did endogenous microglia (153, 155).

The importance of the CNS environment in maintaining the microglial phenotype has been widely demonstrated through transplantation experiments. Primary microglia lose their homeostatic signature within a few hours under cell culture conditions. Genes such as Tmem119, P2ry12, Cx3cr1, Sparc and Sall1 are rapidly downregulated when microglia are exposed to the cell culture environment (157, 158). Conversely, in vitro cultured microglia from both mouse and human regain their homeostatic phenotype once transplanted into the mouse brain in vivo or partially regain them when cultured within brain organoids (157, 159–165). These findings highlight the crucial roles of CNS-derived factors in sustaining the microglial transcriptional program. The nature and cellular sources of these factors are currently under investigation.

### 6.2 The brain borders and the BAM phenotype

Our understanding of whether and how the extra-parenchymal environment shapes the phenotype and functions of BAMs remains limited. Different groups showed that pharmacological depletion of BAMs elicits the recruitment of monocyte-derived macrophages into the brain border niches (112, 166). Similarly, xenotransplantation experiments of human embryonic HSCs into a host mouse brain revealed that these cells have the potential to locally differentiate into BAM-like cells (160, 161). However, it remains unclear whether these BAM surrogates are phenotypically and functionally diverse compared to endogenous BAMs. Nevertheless, identification of macrophage populations phenotypically similar to BAMs in peripheral organs such as lungs, hear, liver, kidney and adipose tissue (121, 123, 167) suggest a conserved transcriptional program presumably instructed by similar environmental cues. Deeper transcriptional and epigenetic analyses are warranted to determine how ontogeny and environmental niches shape the phenotype and epigenetic landscape of BAMs across compartments and conditions.

Epigenetic analyses of BAMs have been hampered by the large number of cells required for the analysis of chromatin accessibility, histone modifications and long-range chromatin interactions. However, bulk low-input and single-cell epigenomic techniques are now becoming increasingly reliable and accessible (168–172). Moreover, techniques to generate iPSC-derived BAMs in vitro or within brain organoids require further optimization and benchmarking. The ability to maintain and genetically manipulate BAMs in culture will offer the opportunity to perform high-throughput single-cell analyses such as CRISPR screens (173). We optimistically expect that in the near future these technologies will be applied to BAMs as well.

## 7 Microglia and BAM in the human brain

### 7.1 Transcriptional signature of human microglia and BAMs

Multiple studies have identified a broad phenotypic overlap between human and mouse microglia under homeostatic conditions (174). For example, human microglia express high levels of CX3CR1, P2RY12, TMEM119, TREM2, MERTK, CST3, NAV3, and SELPLG (94, 175–178). This similarity extends to transcription factor repertoire, as both human and mouse microglia express PU.1, IRF8, MAFB, MEF2C, and SALL1 (158, 175), indicating conserved transcriptional programs across species. Transcriptional profiling of microglia from evolutionarily distant species (ranging from zebrafish, chicken, rodents, monkeys, and human) revealed conserved core signature genes, including Csf1r, P2ry12, Spi1, Irf8, and Tgfbr2 (179), suggesting that such gene expression profile has been positively selected throughout evolution. Nevertheless, human microglia are enriched for several immune-related genes sets which are normally not expressed in mouse microglia under steady state. Genes highly expressed in human microglia but not in mouse include Toll-like receptors, antigen presentation machinery (CD58, HLA-DR, CD74, ERAP2, CIITA), interferon-induced genes (IFI16), chemokines (CCL4), antimicrobial genes (GNLY), complement components (C3), extracellular matrix adhesion molecules (SPP1) (94, 158, 163, 175, 179). Thus, human microglia seem to be prone to immune surveillance and antigen presentation compared to their mouse counterparts. Like microglia, the BAM signature appears highly conserved across species and several marker genes, such as MRC1, F13A1, LYVE1, STAB1, CD163, CD36, MS4A7, CYBB, TGFBI, RBPJ, COLEC12 are shared between mouse and human (178, 180).

### 7.2 Development of human microglia and BAMs

Similar to mice, human microglia and BAMs allegedly originate from yolk sac myeloid progenitors. Recent studies have assessed the transcriptional trajectories of human hematopoiesis throughout embryonic and fetal development (181–184), providing evidence that yolk sac-derived macrophages differentiate into various macrophage lineages across multiple embryonic tissues, including the brain (182). Yolk sac-derived myeloid precursors enter the human embryonic brain through the leptomeninges and the ventricular lumen starting from the 4th gestational week (GW4) (185). Upon infiltrating of the CNS parenchyma, these cells differentiate into microglia, which proliferate and migrate throughout the entire brain. The first peak of microglia expansion occurs around GW9 during the transition from embryonic to fetal stage, followed by a contraction due to apoptosis. A second surge in microglial proliferation occurs during the first postnatal year, reaching a homeostatic microglia density during childhood (185).

Upon seeding the developing human brain, the transcriptional and epigenetic profiles of fetal microglia undergo stepwise maturation until the early postnatal period (186). In the early fetal brain (GW8-9) microglia are enriched for phagocytic pathways and hypoxia-related genes, while immune-related genes (i.e. HLA-DR, C3, ITGAX) are upregulated at later fetal stages (GW17-18) or postnatally (163, 178, 186). This phenotypic maturation is underpinned by significant changes in the transcription factor activity. Transcription factors such as MiTF/TFE, MAF-family, MEF2A, ELF2, E2F2, SOX and SP1 are primarily active in fetal microglia and regulate chemotaxis, cytokine production, adhesion to extracellular matrix, cell cycle and chromatin remodeling. Activity of IRFs, PRDM1, STAT2, KLFs, ETS, MAFB and SMADs increases in postnatal microglia, thus shaping the enrichment for innate immune pathways (186, 187). Likewise, human BAMs undergo a substantial phenotypic maturation across the fetal period gaining expression of HLA, APOE, CD44 and CD163 (178). To date, the transcription factor network orchestrating the development of human BAMs remains poorly investigated.

As described in mice (65–67), human microglia are sustained by a continuous process of apoptosis and cell division, with a daily turnover rate of approximately 0.08%, accounting for about 28% microglia renewal every year, and a medial lifespan of 4.2 years for each microglial cell (188). Assessing the contribution of blood monocytes to the pool of brain macrophages is technically challenging. One study analyzed the frequency of somatic mutations in monocytes and microglia in elderly subjects with clonal hematopoiesis, a condition causing a benign expansion of clonal monocytes carrying mutations in transcriptional and epigenetic regulators. In principle, mutant microglia allegedly derive from monocytes and not from yolk sac progenitors. This study found that 30% to 95% of microglia carried somatic mutations found in monocytes, suggesting BM origin of these cells. Additionally, the clonal size of blood cells strongly correlated with the frequency of mutant microglia, suggesting that monocytes could be a main source of brain macrophages in the aged brain (189). It should however be noted that subjects enrolled in this study were around 80 years old, and age-related medical conditions may alter the balance between yolk sac and BM-derived microglia in the brain. Furthermore, it cannot be excluded that monocytes-derived macrophages carrying somatic mutations may locally undergo clonal expansion at the expense of non-mutant microglia. Another recent study measured the engraftment of monocyte-derived macrophages in the brain of female patients receiving sex-mismatched BM transplant and used the Y chromosome for lineage tracing of BM-derived cells (178). This analysis revealed that about 20% of microglia are BM-derived. Additionally, significant variability in monocyte infiltration was found across different CNS compartments, with a much faster engraftment rate in the choroid plexus as compared to the brain parenchyma, perivascular space and leptomeninges. At the moment, the effect of chemotherapeutic regimen on the turnover of brain macrophages is unknown. The results emerging from these studies suggest that BM contributes to the population of brain macrophages more in humans than it does in mice. Future studies may perform lineage tracing of human brain macrophages across ages and disease conditions using single-cell whole-genome sequencing and random somatic mutations as fate-mapping barcodes (190, 191).

A recent study, currently in preprint, mapped somatic genetic variants at single-cell resolution in microglia and blood to trace monocyte-derived microglia-like cells in the brains of healthy donors and Alzheimer’s patients (192). This study determined that somatic mutations differ substantially between blood and brain, indicating a minor contribution (<5%) of circulating monocytes to the pool of resident microglia. Although still in preprint, this study demonstrates that genome sequencing at the single-cell level is a feasible method to assess the fate of microglia and monocyte-derived macrophages across ages and disease settings.

In summary, the core signature of microglia and BAMs exhibits remarkable similarities in mouse and human, reflecting conserved ontogeny and developmental programs between species.

## 8 Outstanding questions for the field

**Figure 3:**
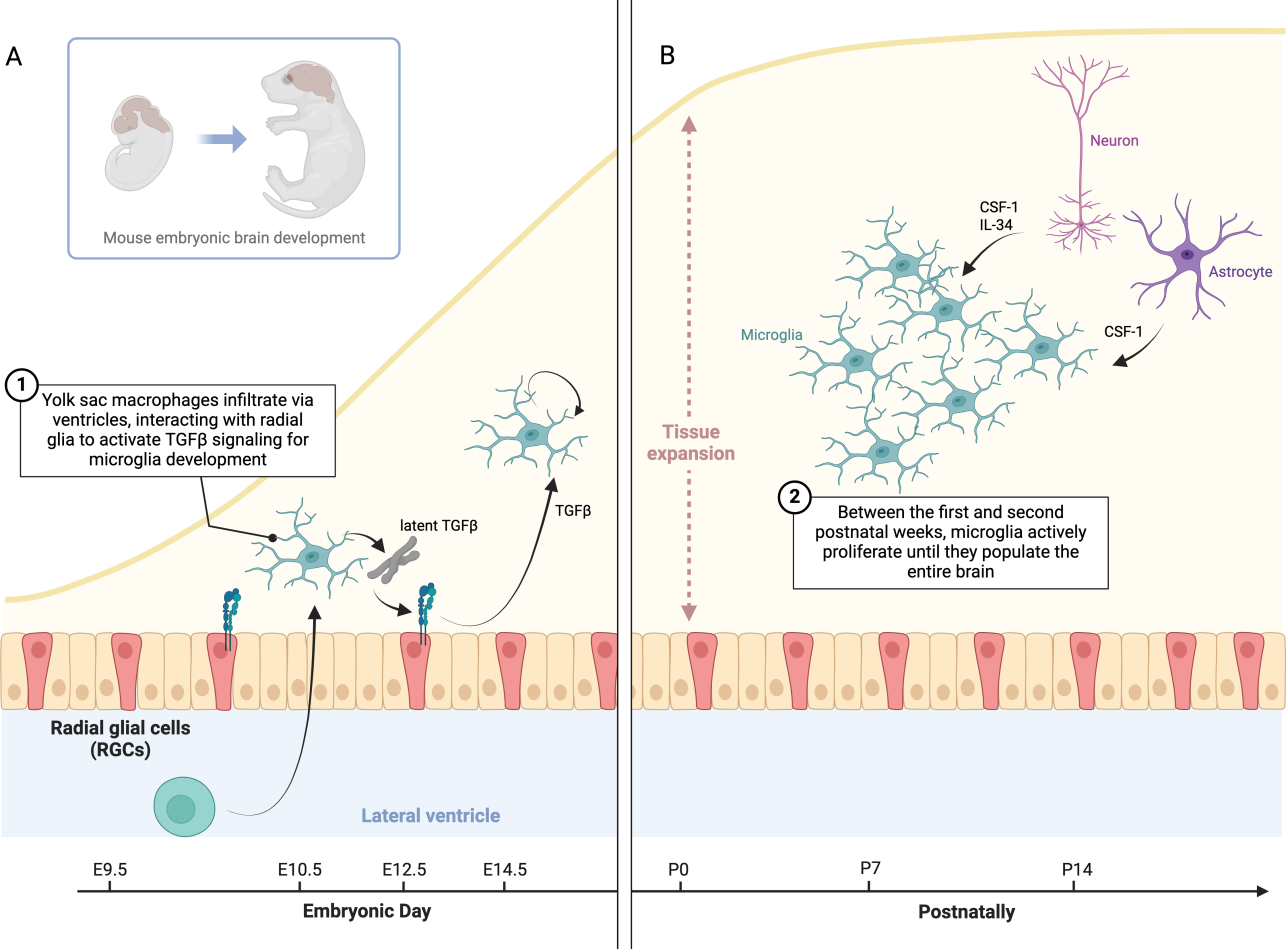
Microglia specification, TGFβ signaling, and proliferation in brain development. **(A)** This schematic illustrates TGFβ signaling in microglia specification and maintenance. During development, yolk sac-derived macrophages infiltrate the brain via the ventricles, where they interact with radial glia to activate latent TGFβ, driving microglial differentiation. Once in the brain parenchyma, microglia rely on autocrine TGFβ signaling to maintain their identity, independent of radial glia. **(B)** Between the first and second postnatal weeks, microglia proliferate to populate the brain, then return to a quiescent state. This raises questions about the timing of proliferation, how microglia density might influence CSF1 or IL34 production, and whether microglia inhibit proliferation upon reaching confluence. Neurons and glial cells may regulate trophic factor expression based on microglial density.

In 2010, the milestone discovery that microglia originate from yolk sac hematopoiesis (26) paved the way for our current understanding of brain macrophages. Despite the tremendous progress made over the past 15 years, several questions remain unanswered. For instance, we now know that microglia specification in the CNS parenchyma critically depends on autocrine TGFβ signaling, which is also required to maintain the microglia signature postnatally. Latent TGFβ secreted by microglia must be captured by αVβ8 integrins to release active TGFβ, which then engages microglial TGFβ receptors in a cell-intrinsic manner. Recent studies suggest that radial glia in the ventricle walls provide αVβ8 integrins to microglial precursors during brain development (104, 107). Therefore, yolk sac-derived macrophages infiltrate the brain via the ventricles, and the interaction with radial glia initiates the primary TGFβ signaling that triggers the microglia developmental program (**Figure 3A**). However, this mechanism raises new questions regarding how TGFβ signaling is sustained as microglia migrate deeper into the brain parenchyma. Once there, microglia are no longer in contact with radial glia, which remain confined to the neurogenic niches. Adult microglia continuously produce latent TGFβ, suggesting that they must rely on autocrine TGFβ signaling via a radial glia-independent mechanism. Although both oligodendrocytes and astrocytes express detectable levels of Itgb8 (103, 107, 193), a specific deletion in these cells does not appear to elicit a remarkable phenotype in microglia (107). Additionally, while αVβ8 and αVβ6 are the most common integrins linked to TGFβ signaling, it remains unclear whether other unknown integrins might be specifically expressed in the brain. This leads us to another open question: do microglia themselves transactivate TGFβ on neighboring microglial cells? Further studies are required to achieve a complete understanding of this mechanism and determine whether it is conserved in humans.

Beyond TGFβ signaling and SMAD4, another key factor in microglia development is the transcriptional regulator SALL1 (99). Mechanistically, SMAD4 binds at the Sall1 super-enhancer, promoting SALL1 expression in microglia. Subsequently, SALL1 recruits SMAD4 to the promoters of microglial genes while repressing BAM genes transcription. The synergy between SALL1/SMAD4 shapes both the epigenetic landscape and transcriptional program of microglia. Yet it remains unclear what epigenetic factors maintain the Sall1 super-enhancer in a poised state in yolk sac-derived brain macrophages. Moreover, we still do not fully understand why other macrophages that experience TGFβ signaling, such as glioblastoma associated macrophages (194), fail to express Sall1.

The complexity of microglia function becomes even more apparent during disease states such as amyloid pathology or demyelination. During this condition, microglia undergo a transcriptional remodeling known as Disease Associated Microglia (DAM), characterized by the downregulation of microglial homeostatic genes and the upregulation of genes involved in innate-immune responses such as Trem2, Apoe, Clec7a, Cd74, Cst7, Spp1, Itgax, Axl (195–198). Interestingly, upon damage recovery, microglia downregulate DAM genes and revert to their homeostatic phenotype (199, 200). This phenotypic transition suggests that during disease conditions microglia dampened TGFβ signaling to mount innate-immune responses. The signals that antagonize TGFβ in microglia during immunological challenge remain unclear.

Mechanisms controlling microglia proliferation is another area of active research. Between the first and second postnatal weeks, microglia actively proliferate until they populate the entire brain parenchyma before returning to a quiescent state soon after (**Figure 3B**). This raises intriguing questions: why do microglia proliferate specifically during this period? Does the abundance of microglia in the brain modulate CSF1 or IL34 production in neurons and glial cells? If so, what are the signals regulating the expression of these genes depending on the microglia density (156, 201). Furthermore, do microglia mutually inhibit cell division upon reaching confluence? If so, what signals mediate this inhibition?

Similarly to microglia, early post-natal proliferation has been observed in PvMΦ (88). However, the factors driving their expansion remain poorly understood, as do the transcription factors and enhancers that regulate the specification of BAMs in brain border regions. Additionally, our understanding of how distinct brain border niches instruct the transcriptional and epigenetic profiles of BAMs, thus shaping their development and specialized functions, remains largely unknown. For example, do BAMs rely on cytokines such as CSF1 or IL34 for their development and survival, or are niche-specific factors involved in the differentiation of the diverse BAM subsets? Furthermore, how PvMΦ communicate with the neurovascular unit remains unclear. Given their role in regulating CSF (119) and cerebral blood flow (137), could PvMΦ also maintain blood-brain barrier integrity, or modulate the severity and progression of cerebral amyloid angiopathy?

Do CpMΦ contribute to CSF protein production or facilitate molecular transport from circulation into CSF? What signals do Kolmer cells exchange with the choroid plexus epithelium under steady-state conditions or during inflammation? Moreover, because these cells express high levels of tetraspanins like CD63 and CD9, markers typically associated with exosome production, do they secrete molecules into CSF via extracellular vesicles?

Lastly, an important consideration is whether these mechanisms are conserved in humans. Several GWAS studies have identified polymorphisms linked to late-onset Alzheimer’s Disease in microglial genes (158, 202–204). However, it remains unknown whether polymorphisms linked to brain diseases map to genomic loci containing BAM genes or enhancers. If so, do these variants affect the development and function of human BAMs? Hence, can we target BAMs to treat neurological disorders?

While significant strides have been made in deciphering the roles of brain macrophages like microglia and BAMs, many questions remain unanswered. We foresee that future research will help shed light on these open questions, further expanding our understanding of the intertwined relationship between brain and immune system.

## 9 Conclusion

With this review, we discuss the molecular mechanisms and environmental factors that shape the development and specification of brain macrophages within their tissue niches. Both microglia and BAMs exhibit substantial phenotypic similarities between mice and humans, suggesting that their developmental trajectories could be conserved across species. Although several aspects of their biology remain obscure, advances in single-cell omics and single-cell perturbation techniques continuously push forward the boundaries of our knowledge. Future interdisciplinary research will delineate more precisely how brain macrophages communicate with other brain cells and regulate brain physiology in health and disease. The long-term goal of this line of research is to develop approaches to target and manipulate microglia and BAMs for therapeutic applications in humans. Our research efforts have made tremendous progress in the past decade, encouraging optimism for upcoming scientific achievements.
